# How well prepared are hospitals for future crises? Board members perceive their hospitals as resilient for acute crises

**DOI:** 10.1186/s12913-024-11197-4

**Published:** 2024-07-16

**Authors:** Caroline Schlinkert, Laura Muns, Lilian van Tuyl, Cordula Wagner

**Affiliations:** 1 Nivel (Netherlands institute for health services research), Otterstraat 188, 3513CR, Utrecht, The Netherlands; 2 VUmc University Medical Centre, De Boelelaan 1117, 1081HV, Amsterdam, The Netherlands

**Keywords:** Patient safety, Quality of care, Complex adaptive system, Resilience, Crisis management

## Abstract

Resilience is an organizational capacity in day-to-day practice and crisis situation performance. A one of a kind crisis for hospitals is the COVID-19 pandemic. The long duration and magnitude of this crisis offers the opportunity to gain insight into the complexity of crisis management and organizational resilience of hospitals. This interview study therefore explored the organizational resilience of Dutch hospitals during the first 14 months of the COVID-19 pandemic. Nine board members of nine Dutch hospitals were interviewed by means of a semi-structured interview that was built on thirteen indicators of organizational resilience. The results showed that board members considered their hospitals as resilient on almost all indicators. Their judgments varied about how prepared and ready for future crises they considered their hospital. According to board members, hospitals are mainly prepared for “acute” short-term crises, thanks to good crisis leadership, open communication and strong networks. A crisis as long as the COVID-19 pandemic was unprecedented and therefore more difficult to deal with. In between the infection waves, work processes were reflected upon to learn, anticipate and respond more smoothly to successive waves. However, the enduring nature of the COVD-19 crisis presented complex organizational challenges. Crisis operations were eventually scaled down and hospitals had to manage the crisis and regular care as two companies side by side. Each crisis manifests differently. Fostering trust in healthcare staff and allowing them to act autonomously during crises, while diligently monitoring external influences and potential future crises, are therefore paramount in developing organizational adaptive capacities.

**Supplementary Information:**

The online version contains supplementary material available at 10.1186/s12913-024-11197-4.

## Background

Healthcare systems have experienced major crises over the past two decades. For instance, the 2008 global economic crisis, the 2014–2016 Ebola outbreak on the African continent and the COVID-19 pandemic [1, 2]. These crises and disasters destabilize health organizations and their employees on personal, social and organizational aspects for a longer period of time [3]. The COVID-19 pandemic and the previous health crises have therefore catalyzed the attention to the concept of resilience in the global health discourse [4, 5]. Resilience in healthcare, especially in hospitals, is essential as hospitals provide ‘lifeline’ services to minimize the impact of disasters on the community [6]. Resilience in hospitals will therefore be examined in the present study.

Resilience refers to how an organization can handle both everyday tasks and crisis situations [7]. Resilience in healthcare organizations may lower their chances of being affected by crises and can be helpful in dealing with future emergencies [8]. This is because resilient healthcare organizations can not only handle crises but also learn from them, identify risks and vulnerabilities, and prepare for a future where unexpected events have less severe consequences [3, 9]. At a broader level, resilient healthcare organizations can also indirectly help communities recover more quickly from emergencies [10]. This is because communities rely on essential services provided by organizations like (hospital)healthcare facilities, as well as power, water, and transportation services, to respond to and recover from crises.

Resilience is as such traditionally viewed as a matter of crisis or emergency management [11]. More recent research focuses on the complex interplay between building resilience for everyday operations and ensuring readiness for crisis response and recovery. Most of this work is so far non-empirically examined by different scholars around the world [11, 12]. Nevertheless, there is significant growth in empirical research, primarily consisting of observational studies. These endeavors demonstrate the importance of gaining a thorough understanding of everyday clinical work, often referred to as “work-as-done.” [12].

That resilience matters for hospitals has emerged prominently with the rise of the COVID-19 health crisis. This crisis created abrupt challenges for hospitals, because it required them to simultaneously plan for and manage a rise of COVID-19 patients, while also maintaining daily, essential health services. Countries worldwide differed in their approach to deal with the crisis, due to differences in healthcare capacities, political leadership and material supply [13, 14]. A recent review across 16 European countries also found commonalities. Many countries (including the Netherlands) initially hospitalized COVID-19 patients, but transitioned to outpatient care as much as possible as the pandemic progressed. Additional to more home care, cancelling or postponing treatments were common adaptations as well [15]. Overall, it was concluded that increasing the IC capacity was vital to have enough volume to the increased IC demand.

The abrupt care system changes as well as the long duration and magnitude of the present COVID-19 health crisis offer the opportunity to gain insight into the complexity of the day-to-day organizational resilience and crisis management of hospitals. Hospitals may struggle to meet the sudden increased demand for care due to staff and resource shortages. Hospital leaders must therefore find ways to overcome these operational challenges to ensure that patients receive the care they need. At the same time, hospital leaders need to find novel approaches to plan, respond to, and manage the crisis [16]. The approaches and management strategies adopted by hospital board members at the onset of the COVID-19 pandemic may provide valuable insights into resilient organizational performance, offering lessons that can inform our response to future crises. The present interview study therefore aimed to explore the strengths and weaknesses of the organizational resilience of Dutch hospitals during the first part of the COVID-19 crisis by interviewing hospital board members.

## Materials and methods

### Participants and procedures

Ethical review and approval were waived for this study, because it was an online interview study asking for information that was already being tested for ethical approval in another study on organizational resilience (2022.0088, The Medical Ethics Review Committee of VU University Medical Center) [17].

We recruited nine hospital board members from nine different hospitals (two academic, four top clinical, and three general) to participate in our study. The participants were drawn from a pool of board members of 20 Dutch hospitals that had also participated in a broader, national study on patient safety and preventable adverse events in hospital care [17]. Semi-structured interviews were conducted with the hospital board members between April to December 2021 by two of the authors (C.S. and L.M.) who have academic backgrounds in psychology and anthropology.

Due to the COVD-19 restrictions at this time, the interviews took place via video call.

During the video call, participants were first informed about the study content and had to verbally agree to the study conditions. It was indicated that the results of the study would be anonymized. Participants were encouraged to give honest and critical answers. The interviews lasted between 45 and 60 min and were videotaped and transcribed verbatim. Data saturation was reached when no new themes or insights emerged from the interview data. To prevent researcher misinterpretation, all participants received and approved their interview summaries. Two authors (L.M. and C.W.) independently read and analyzed all interviews using thematic content analysis [18]. The findings were compared and discussed by L.M., L.T. and C.W. at each stage of the analysis.

### Interview structure and Benchmark Resilience Tool

**Fig. 1:**
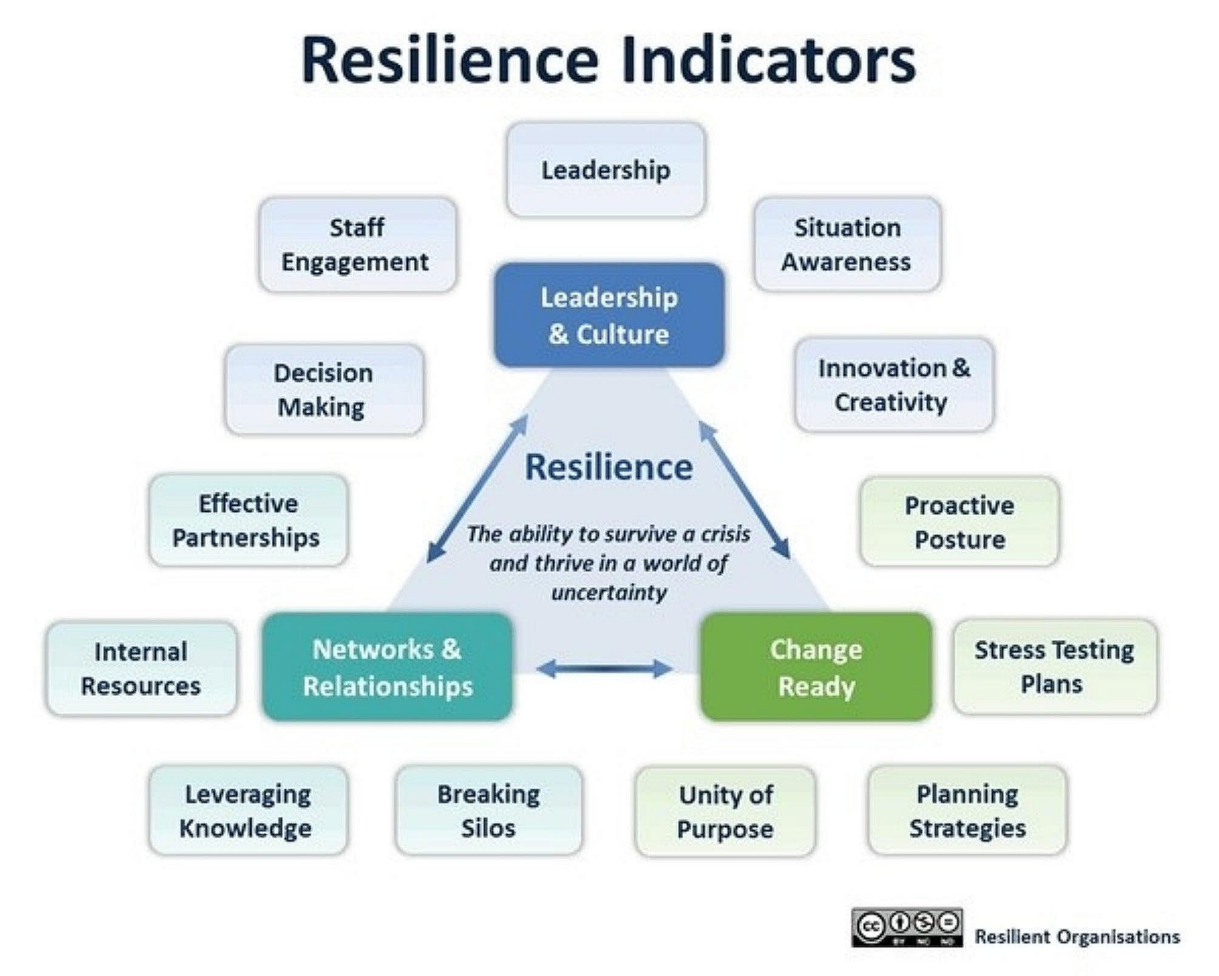
The thirteen resilience indicators as found on resorgs.org.nz

Organizational resilience as defined by Seville and colleagues [9] is found to have thirteen indicators that can be summarized in the three principal themes: A leadership and culture that fosters flexibility and adaptability of employees, having strong networks and relationships to fall back on in crisis situations, and being future change ready to anticipate to the unknown [9, 19]. For a visual overview of all indicators please consider Fig. 1. These indicators were developed by Seville and colleagues based on a grounded theory approach to discover what leads to resilience in an organization [10]. We explored the status quo of organizational resilience among Dutch hospitals based on these thirteen indicators [9], and examined what had changed in organizational processes during the first waves of the COVID-19 crisis through further questioning.

The interviews had a semi-structured nature that were based on the Benchmark Resilience Tool – short form [20](BRT). The interview script was specifically developed for this study and can be found in Appendix B. The BRT questionnaire assesses behavioral traits and perceptions that belong to the theoretical constructs of organizational resilience (Fig. 1). It comprises thirteen statements that each represent one organizational resilience indicator. Example statements are: “We are mindful of how a crisis could affect us” and “Our organization maintains sufficient resources to absorb some unexpected change”. The 13 indicators can be further divided into two latent factors “planning resilience” (example question 1) and “adaptive capacity” (example question 2).

Interview leaders (C.S. (PhD in Psychology) or L.M. (MA in Anthropology) read aloud the meaning of each theme and corresponding indicator for participants). For each BRT statement, participants then gave an oral assessment on a scale of 1 to 5 (strongly agree, agree, neither agree/do not disagree, disagree, completely disagree). Through this statement, they consequently reflected on their experiences during the COVID-19 pandemic in their own hospital. Participants were additionally encouraged to envision the future of their organization, post-COVID-19. During the whole interview, we checked whether de interview partners understood all statements well and encouraged the interviewees to ask questions when there were unclarities. All reflections were qualitatively analyzed within their themes and summarized via the program MaxQDA [21]. Appendix A contains the steps of analysis.

The BRT instrument has not been previously validated in the Netherlands. To ensure cultural and linguistic equivalence, the thirteen BRT indicators and corresponding statements were translated using a forward-backwards translation procedure.

## Results

### Participant characteristics

Participants were between 54 and 65 years of age (M = 58 years). Of these, four were women and five were men. Their working positions were chairman of the executive board (*n* = 6), member of the executive board (*n* = 1), chairman of the council committee (*n* = 1) and chairman of the crisis policy team (*n* = 1). Participants were employed in their working sector between 15 and 40 years (M = 27 years) and were employed at the current hospital between 5 months and 32 years (M = 8 years). Participants typically hada medical background with previous experiences in policymaking.

### Benchmark Resilience Tool outcomes

The mean score on the BRT questionnaire was overall high (M = 4.2; SD = 0.41). The three themes “Leadership and culture” (M = 4.3, SD = 0.21), “Networks and relationships” (M = 4.2, SD = 0.65) and “Change ready” (M = 4.0, SD = 0.33) showed similar results. Moreover, the two BRT subscales “planning” (M = 4.2, SD = 0.44) and “adaptive capacity” (M = 4.2, SD = 0.42) were likewise high. Hospital board members thus judged their hospital as resilient. The interviews offered deeper insights into why hospital board members had a positive outlook, while also highlighting the challenges they faced in maintaining resilience.

### Interview outcomes

We summarize below the hospital board members’ answers per resilience theme “Leadership and culture”, “Networks and relationships” and “Change ready”. Practical examples supplement the summary where applicable.

#### Leadership and culture

*Indicator 1: Leadership.* The team leaders were evaluated very well by the board members. Discussions were held about policy consistency, keeping calm in stressful times, supporting employees and correctly prioritizing what is important and urgent. Also, organization-wide responsibility and decision making was seen as important. This means that not only affected departments had to solve problems related to the pandemic, but the organization as a whole was responsible. According to board members, employees had the confidence that they and the situation were being watched, and that they could fall back on their supervisors. One interviewee indicated that the structure of the crisis policy team (CPT) and the operational policy (OP) team contributed greatly to streamline responsibilities and transparency within the organization, which increased trust among staff.

During the first infection wave in which there was an acute crisis, all focus was on COVID-19. During the second infection wave and the period after that, it was decided to continue regular care as far as possible. This however made organizing and leading the organization more complicated. The board members spoke of a dichotomy in the organization. By dichotomy was meant running two companies side by side: a crisis company with accompanying leadership in the form of a CBT and OT and also running a regular company, so that regular processes within the hospitals could also take place.

*At some point it takes a very long time. That does something to the people and the organization and you have to organize accordingly. We also deliberately stopped the crisis policy very early, […] And we transferred that to a task group. With the idea of: it’s back to normal and there are other things too. You cannot allow that hospital to function month after month in such a crisis regime.*

*Indicator 2: Staff engagement.* According to the board members, staff took responsibility independently and on their own initiatives. Staff members who normally did not present themselves this way showed leadership qualities. By giving staff members the freedom to act autonomously their skills were used to create a more resilient organization during this crisis. Interviewees indicated that the sense of responsibility of the staff was greater than in non-crisis times and that staff had to deal with many unexpected situations. For instance, in nursing wards that became COVID-19 wards, nurses took the lead and autonomously made decisions about the safe furnishing. At the reception, staff had to quickly find a solution for aggression from visitors due to a visit ban during the first infection wave.

*What we have learned in this crisis is that basically permanent communication from the CBT-OT to all employees in the organization […] whether they are involved in the crisis or not. This is of decisive importance to motivate people for the job they are in.*

*Indicator 3: Situation awareness.* According to the board members, the organization and employees closely monitored on what they were doing. Hence, whether it was safe and whether it was going well. Due to the close monitoring, processes and care provision could often remain at the same level as before the pandemic.

*Indicator 4: Decision making.* Interviewees indicated that the input of staff was valued during the crisis moments, because they had the most knowledge based on their expertise. Although the final decision was made with the chairman of the crisis team, this decision was often taken with the knowledge and input of all stakeholders.

*What normally preceded a whole meeting situation could now be discussed in two days. Both between internal and external partners. As for internally, I can give an example about housing internal medicine and pulmonary medicine in the ward with the most isolation rooms. They have been talking about this for years and now they are there and they are not going away.*

*Indicator 5: Innovation and creativity.* Especially during the first infection wave, situations arose that required immediate action. This often manifested itself in creative solutions. Departments were made “COVID-19-proof” by for instance putting up perplexed screens. One hospital had devised a system of hatches in departments that were not suitable for COVID-19, with an official behind it who received things from one side and passed them on to the other side. Creative solutions were also found for the shortage of protective equipment. For example, one hospital had bought diving masks from a sports shop, an idea suggested by the internists and supported by the crisis management team. Moreover, several interviewees indicated that the transition to digital care had taken off in the COVID-19 crisis, and that this is very likely a permanent change. Especially during the first infection wave, many consultations had to be held remotely by means of video calling or the use of eHealth apps.

#### Networks and relationships

*Indicator 6: Effective partnerships.* The interviewees liked the enhanced cooperation with for instance general practitioners and hoped that the intensified relations would continue after the COVID-19 crisis. One interviewee indicated that there was already a good relationship with the general practitioners (GP) before the crisis. It was emphasized that this relationship has been very important during the crisis. Due to the limited number of beds, more patients needed to be looked after by the GP. Collaboration with GPs was thus needed to relieve the burden on hospital staff as much as possible.

*When we knew a lot more about corona, we also arranged together with the general practitioners and the pulmonologists that people could go home much faster with a certain oxygen pump, so that we relieved the hospital again, but also prevented people, especially the elderly, from having to go to hospital.*

New work processes between caregivers were set up during the crisis, some of which will be maintained. An example is a new information system to promote patient information exchange between general practitioners and hospital specialists. Organizations also looked at the possibility of treating other groups of patients at home (besides COVID-19 patients).

Several interviewees emphasized that there was mainly collaboration with the National Acute Care Network, including collaborating with the regional acute care organs. Some interviewees said that a number of new partners had been added to this existing network in the context of the crisis, for example disability care and local healthcare centers. Under normal conditions, consultations between the hospital and the national network take place three times a year. At the beginning of the COVID-19 crisis, the partners involved consulted on a daily basis. This was later scaled down. Moreover, there was consultation between regional hospitals about bed capacities, IC occupancy, intake from general practitioners and with (home)care institutions. Information was also exchanged regarding the availability of protective equipment and solutions were jointly sought for shortages of these. Hospitals also took up the coordination of protective equipment for other healthcare institutions.

*Indicator 7: Leveraging Knowledge.* The interviewees indicated that the provision of information was generally good. However, at some point there was incidentally information overload, because many new work processes got introduced very quickly. Due to this enormous influx of information, hospitals chose to communicate information digitally to healthcare staff. For example, a video was recorded explaining decisions and new protocols. The use of the videos was very well received by staff. Several interviewees therefore indicated that communication via video might be something that in the future, albeit much less intensively, may be retained for sharing important information.

*Indicator 8: Breaking silos.* Interviewees indicated that the group spirit and camaraderie was greatest during the first period of the pandemic. One of the interviewees described the feeling during the first phase among staff and management as an “*acute euphoria phase*”, in which everyone picked up things very quickly due to the stress-related adrenaline release. However, as the crisis continued, there was less and less a sense of “*we can do it*”. As the COVID-19 infections decreased, healthcare workers started thinking about how to proceed with their own patients and department. Some were concerned about whether, for example, they would have to work in the COVID-19 department again if the infections would rise again, and were afraid of this. Over time, there was also friction between running a crisis company and a regular company side by side, and resistance to solve the problems of other departments or hospitals increased.

*Everyone is always focused on situations related to COVID-19, which leads to a great sense of community and shared focus. What I am less enthusiastic about is the things that are left behind, and that less attention is paid to them. It does have a downside. Because an increase in waiting lists also simply leads to problems. Waiting list management, which normally always happened routinely and never got out of hand, is now getting out of hand.*

*Indicator 9: Internal resources.* Answers to this indicator varied. Some interviewees indicated that their hospital had been short in protective equipment and staff, but were financially well set up. Other organizations highlighted the precarious financial situation as a main problem. Other resources that had been lacking were ER capacity, information exchange and adequate staffing. Due to a lack of staff, one interviewee even said that his hospital had to “*close the front door”* (which means temporary admission halt) a number of times. Similarly, another interviewee explained that short-term upscaling of staff was no problem, but that it was difficult to scale up for long periods. Opinions were divided on how to solve this problem. Some interviewees stated that there was already a shortage of staff before the COVID-19 crisis. They argued for a less lean organization of hospitals to increase flexibility within the organization. The interviewees emphasized that the current Dutch healthcare system has a low capacity to admit new patients. The gap left by departing or ill staff was seen as difficult to fill because “*these staff simply aren’t there*”.

#### Change ready

*Indicator 10. Unity of purpose.* Interviewees found this statement difficult to answer. This was mainly because they indicated that the crisis was not over yet and that they were still very busy managing and solving crisis-related situations. It was also indicated that the COVID-19 peak may have passed, but that the pressure on care remained high. Recovery was not yet a priority. One interviewee explained that COVID-19 care only accounts for 10% of healthcare in the Netherlands. The other healthcare remained on very high strain and the pressure on the healthcare system would probably only increase due to future catch-up care.

*In September of 2020, we started a process to draw up a new strategic policy plan. […] We also said that we do this without outside help. And that must be ready so that people can include this in the new plans […] We succeeded, it has become a very nice plan. So we were lucky enough to be able to work together on that new strategic plan in the second part of that crisis period. And that has given a lot of bonding, the focus, unambiguity in what we have to do with the elements that float to the surface in that plan, that is very large.*

*Indicator 11. Proactive posture.* According to the board members, hospitals are good at reacting quickly to unexpected events. An interviewee compared a university hospital with a large and cumbersome cruise ship that moves slowly, but emphasized that in times of crisis it was possible to switch gears very quickly. Interviewees indicated that there was already a certain basic training among staff and management with regard to crisis response. Due to the complexity of care, hospitals are used to deal with great variation on a daily basis. It was also stated that the staff and organization were at their “*very best*” during the first wave. However, proactiveness declined due to the length of the crisis.

*Hospitals are of course used to dealing with a lot of variation. If you look at what enters the hospital and how it flows through the hospital; that’s not uniform at all. […] we are constantly working to anticipate individual changes. And therefore, in principle, it also makes us alert in responding to changes at the system level.*

*Indicator 12. Planning strategies.* Before the COVID-19 pandemic, a crisis of this magnitude and duration was not foreseen. Prior training was mainly for short-term crises and disasters. An interviewee indicated that as an organization, they had learned from the COVID-19 crisis and had therefore adjusted their crisis response strategy. Plans were rewritten on the basis of current evaluations. Procedures that worked less well were adjusted. Training was also renewed and new training courses were developed based on the new knowledge. Several interviewees emphasized that there is now more awareness for future virus outbreaks and pandemics.

*We have made agreements […] about what level of COVID care can be resolved […] without affecting the rest of the hospital. […] We think that the COVID problem will remain with us for years to come.*

*Indicator 13. Stress testing plans.* It was explained that the hospitals have a standard protocol for stress testing plans. A number of employees are thus specifically trained in roles such as crisis coordinator. Board members also go through these training cycles. However, some interviewees admitted that these trainings were not regularly put into practice in their organization. It was explained that there are protocols and scenarios ready for certain crises, but that these are not always practiced with the staff.

*[…]not every crisis comes in the same way. Planners want to plan certainty and only look at the previous crisis and then do a lot of things to manage the same kind of crisis again, while the next crisis becomes another one. That makes little sense. […] plans that have always been made are really not pulled out of the closet. And often they just don’t work anymore. […] you can also arrange things well together at that moment.*

## Discussion

The current study investigated organizational resilience within nine Dutch hospitals during the initial phase of the COVID-19 pandemic in 2020/2021. Overall, hospital board members assessed their hospitals as resilient based on the Benchmark Resilience Tool. However, semi-structured interviews revealed that hospitals were primarily equipped and trained to handle short-term acute crises. This emphasis was evident in the positive ratings given by board members for the first two themes of organizational resilience: leadership and culture, and networks and relationships. Frequent rehearsal of crisis scenarios enabled the hospitals to respond quickly and effectively to the first two waves of COVID-19, thanks to good crisis leadership, open communication, and strong networks.

In between the infection waves, work processes were reflected upon to learn, anticipate, and respond more efficiently to the successive waves. However, the persistence of this crisis presented complex organizational challenges, as shown by the more mixed answers in the third theme: change readiness. Resilient capacities of the hospital staff gave way to anxiety, exhaustion, and increased absenteeism. At a certain point, the crisis organization was scaled down and hospitals now have to manage the crisis and the regular care as two companies side by side.

Against the instinctive response to tighten control in times of crisis and uncertainty, our study revealed that a resilient crisis response benefits from a structured release of control to increase staff autonomy [9, 22, 23]. (Personal) Autonomy can be defined as being able to act free of controlling influences and with the capacity for intentional action [24]. An example of (personal) autonomy can be seen in the results of indicator 2, staff engagement. When staff members were given more freedom to make decisions, they could use their skills to handle new situations and even show leadership qualities they might not have shown before. It is important for this behavior to show that staff members trust their supervisors. According to the board members we talked to, this trust was in place, as shown in the results of indicator 1, leadership.

Our findings herewith echo other research that showed that the ability to anticipate, resist and respond to crisis depends on leaders’ knowledge of context specific factors and their understanding of the work setting [25, 26]. Indeed, improvisational behavior was stimulated from the board members and hospital team leaders by relying on unique staff knowledge. Examples of successful improvisational changes are described at indicator 6, effective partnerships, like the new information system to promote patient information exchange. These changes were inspired by this specific crisis and will therefore help create a more resilient organization than detailed crisis plans that were written based on past experience, as mentioned at the results of indicator 13.

The employees with the best situational knowledge received more responsibilities, regardless of their position within the organization. Moreover, effective networks with primary care proved to be very important to manage patient outflow as much as possible, to maintain IC capacity [15]. However, to avoid chaos, leaders provided a framework within which innovation and decisions could take place. The value that was given to the personal autonomy of staff members had fostered a sense of togetherness and mutual trust within the organization [9, 22]. Through timely and complete communication from management to employees and mutual feedback, employees felt seen in their questions and uncertainties. Digital communication technology proved hereby effective.

Crises have become increasingly complex [27]. This complexity is also demonstrated by the aftermath of the COVID-19 crises. Transition to the ‘new normal’ in which COVID-19 is part of healthcare poses a challenge. Due to the postponed regular care, hospitals are dealing with a catch-up crisis. In September 2021, it was determined that between 170,000 and 200,000 operations still need to be performed in the coming months in the Netherlands [17, 28]. Such ‘creeping’ crises are likely to ‘simmer’ long after the crisis peak has passed [29]. This phenomenon was already seen before COVID-19. It is usually the result of a short-term view in response to the acute crisis, which causes the situation to worsen or simply be displaced [29].

The question of how to steer healthcare organizations, such as hospitals, to anticipate future unknown crises is an urgent one that needs to be answered by the leaders within healthcare organizations, as well as their political, societal and financial networks. It is of course impossible to anticipate and prepare for all crises. Unforeseen crises thus require a distinctive leadership response that often includes being flexible and adaptable, making good decisions quickly, and mustering resources on short notice [27]. The COVID-19 health crises and its aftermath permit a collective effort to research, teach, and develop such good crisis leadership.

This study examined hospital board members’ perceptions of organizational resilience during the early COVID-19 pandemic. The mixed interview methodology employed in this study is a strength, as it allowed us to collect both ratings and experience data. We report on concrete resilience ratings that are supported and contextualized by qualitative quotes from board members. Another strength of this study is that the interview structure was based on existing theory of organizational resilience [9]. This allowed us to draw on existing knowledge of resilience to interpret the board members’ responses in a theoretically grounded and systematic manner.

A limitation of this study is that we cannot generalize the findings to all healthcare staff, as their experiences may differ from those of board members. Healthcare staff are on the front lines of patient care, and as such, they have a unique perspective on the challenges and opportunities that hospitals face [30]. Also, healthcare staff are a diverse group, and their opinions on organizational resilience may vary. This is due to a number of factors, such as their job role, level of experience, and the specific challenges of the hospital where they work [31]. Future studies should thus focus on the perspectives of healthcare professionals on organizational resilience to expand and verify the current findings. Additionally, other healthcare domains, such as primary healthcare and home care, are also interesting research fields to study organizational resilience, allowing for comparisons with the present results [32].

Further, our findings are limited to the Dutch health care system. Recently, the Benchmark Resilience Tool was administered among healthcare managers in two Turkish hospitals [33] and among long-term care leaders in North Carolina, United States [32]. In the Turkish study, the hospital management staff rated the hospitals as overall resilient, a finding that is similar to ours. However, the highest rated indicators were ‘planning strategies’ and ‘stress testing plans’, indicators that belong to the theme ‘change readiness’. Lowest ratings were given to ‘innovation and creativity’ and ‘internal resources’, indicators that were rated high in our study. However, again similar to our findings, the North Carolina study found that the indicators belonging to ‘change readiness’ were rated lowest among their sample and ‘leadership’, ‘staff engagement’ and ‘situation awareness’ were rated highest. They interpreted their findings as a pandemic-related lowered experienced of planning strategies skills. Future studies need to clarify how these international discrepancies evolved and how the different healthcare systems can learn from each other to organize hospital care resiliently. More general, resilience researchers have thus far made important contributions to conversations on how to enhance the provision of healthcare [34]. However, investigating resilience in healthcare is still easier said than done. The biggest challenges for this research field lie in creating clarity about the resilience concept, as well as interdisciplinary collaborations with corresponding methodologies to study the complexity of resilient healthcare.

## Conclusions

The COVID-19 pandemic contributes to the awareness of how crises can destabilize health organizations and their employees for a longer period of time. To act resilient as healthcare organization in crisis, this study shows that it is important to trust the healthcare staff and let them act autonomously while also monitoring external influences and their associated future crises and threats. The latter to learn to anticipate future crises and therefore be more resilient as an organization. Organizations are in a macro-environment in which they are influenced by societal, economic and political decisions and developments. The implementation of lessons learned by the COVID-19 pandemic will therefore depend on these societal structures to enhance organizational resilience in healthcare in the future.

### Electronic supplementary material

Below is the link to the electronic supplementary material.

Supplementary Material 1

Supplementary Material 2

## Data Availability

The data supporting the conclusions of this article are included within the article and its additional files. Raw data can be requested from the corresponding author.

## References

[1] Kruk ME (2015). What is a resilient health system? Lessons from Ebola. Lancet.

[2] Thomas S (2013). A framework for assessing health system resilience in an economic crisis: Ireland as a test case. BMC Health Serv Res.

[3] Kuntz JC (2021). Resilience in Times of Global Pandemic: Steering Recovery and thriving trajectories. Appl Psychol.

[4] Haldane V (2017). Health systems resilience: meaningful construct or catchphrase?. Lancet.

[5] Kruk ME (2017). Building resilient health systems: a proposal for a resilience index. BMJ.

[6] Zhong S (2014). Development of hospital disaster resilience: conceptual framework and potential measurement. Emerg Med J.

[7] Seville E (2008). Organisational resilience: researching the reality of New Zealand organisations. J Bus Contin Emer Plan.

[8] Parsons D. *National organisational resilience framework workshop: The outcomes*. in *National Organisational Resilience Framework Workshop*. 2007.

[9] Seville E. Resilient organizations: how to survive, thrive and create opportunities through crisis and change. Kogan Page; 2016.

[10] McManus S (2008). Facilitated process for improving organizational resilience. Nat Hazards Rev.

[11] Boin A, McConnell A (2007). Preparing for critical infrastructure breakdowns: the limits of Crisis Management and the need for Resilience. J Contingencies Crisis Manag.

[12] Hollnagel E, Braithwaite J. Resilient health care. CRC; 2019.

[13] Unruh L (2022). A comparison of 2020 health policy responses to the COVID-19 pandemic in Canada, Ireland, the United Kingdom and the United States of America. Health Policy.

[14] Chen Y-Y, Assefa Y (2021). The heterogeneity of the COVID-19 pandemic and national responses: an explanatory mixed-methods study. BMC Public Health.

[15] Webb E (2022). Providing health services effectively during the first wave of COVID-19: a cross-country comparison on planning services, managing cases, and maintaining essential services. Health Policy.

[16] Mohtady Ali H (2023). Enabling transformational Leadership to Foster Disaster-resilient hospitals. Int J Environ Res Public Health.

[17] Caroline Schlinkert SvS, Jelsma J, van Schoten S, Wimmer P, Kroeze M, Wagner C. *De Resilience Analysis Grid (RAG) in Nederlandse ziekenhuizen: Ontwikkeling, evaluatie en eerste conceptversie van een Safety-II reflectie tool* 2023.

[18] Clarke V, Braun V (2017). Thematic analysis. J Posit Psychol.

[19] Lee AV, Vargo J, Seville E (2013). Developing a tool to measure and compare organizations’ resilience. Nat Hazards Rev.

[20] Whitman R (2013). Short-form version of the Benchmark Resilience Tool (BRT-53). Measuring Bus Excellence.

[21] Oliveira M et al. *Thematic content analysis: Is there a difference between the support provided by the MAXQDA® and NVivo® software packages*. in *Proceedings of the 12th European Conference on Research Methods for Business and Management Studies*. 2013.

[22] Lloyd-Smith M. The COVID-19 pandemic: resilient organisational response to a low-chance, high-impact event. Bmj Leader; 2020. 10.1136/leader-2020-000245 37579283

[23] Lombardi S, Cunha MPe, Giustiniano L (2021). Improvising resilience: the unfolding of resilient leadership in COVID-19 times. Int J Hospitality Manage.

[24] Beauchamp TL, Childress JF. *Principles of biomedical ethics*. Eighth edition. ed. 2019, New York, NY: Oxford University Press.

[25] Fagerdal B (2022). Exploring the role of leaders in enabling adaptive capacity in hospital teams – a multiple case study. BMC Health Serv Res.

[26] Uhl-Bien M (2021). Complexity and COVID‐19: Leadership and Followership in a Complex World. J Manage Stud.

[27] Riggio RE, Newstead T (2023). Crisis Leadership. Annual Rev Organizational Psychol Organizational Behav.

[28] Zorgautoriteit N. *Oplegger Monitor Toegankelijkheid van Zorg; gevolgen van Covid-19-26 augustus 2021 [Internet], 2021. Report No: PUC_652707_22*.

[29] Boin A, Ekengren M, Rhinard M (2020). Hiding in Plain Sight: conceptualizing the creeping Crisis. Risk Hazards Crisis Public Policy.

[30] Rosted E (2021). On the frontline treating COVID-19: a pendulum experience—from meaningful to overwhelming—for Danish healthcare professionals. J Clin Nurs.

[31] Hølge-Hazelton B et al. The differences in experiences among multi-level healthcare leaders, between the first and the second wave of the COVID-19 pandemic: two cross-sectional studies compared. J Healthc Leadersh, 2021: p. 209–19. 10.2147/JHL.S326019 PMC8445098 34539192

[32] Lane S et al. *Self-Reported Resilience of North Carolina Long-Term Care Organizations and Public Health Agencies in the Midst of the COVID-19 Pandemic* Available at SSRN 4172861, 2022.

[33] DOĞRUSÖZ LA, YAZICI S, OMAY EGG (2022). Organizational Resilience in Healthcare Organizations: A Research in the Public and Private Sector. Afet ve Risk Dergisi.

[34] Smaggus A, et al. Investigating resilience in Healthcare: easier said than done? A response to the recent commentaries. International Journal of Health Policy and Management; 2022. 10.34172/ijhpm.2022.7682 PMC10125065 37579451

