# Supplementary material for: How well prepared are hospitals for future crises? Board members perceive their hospitals as resilient for acute crises
Source: BMC Health Serv Res. 2024 Jul 16;24:817. doi: 10.1186/s12913-024-11197-4 (PMC11251290; doi:10.1186/s12913-024-11197-4)
Supplement: Supplementary file 1 — Supplementary Material 1 [file 12913_2024_11197_MOESM1_ESM.docx]

**Appendix A**

**The steps of the thematic analysis as performed during this research:**

| **Step** | **Definition** | **Author** | **Activities** |
| --- | --- | --- | --- |
| Step 1 | Conducting the interviews | C.S. and L.M. | Online interviews with hospital board members |
| Step 2 | Reading and re-reading interview transcripts | L.M. | Recording first thoughts about the data and salient remarks |
| Step 3 | First round coding in MaxQDA | L.M. | Coding the data from the salient remarks in step 1  Coding the data along the BRT indicators  Writing memos with the data |
| Step 4 | Testing and falsifying findings | L.M. en C.W. | Organize and present coding. Test the findings together with the second researcher, per theme/indicator:   - Does the data support the themes found? - Are there overlapping themes? - Are there sub-themes? - Do the themes correspond with the indicators? |
| Step 5 | Defining and interpreting themes | L.M., L.T. and C.W. | Search for examples/quotes that are representative of the themes/indicators. The overarching themes identified therein were subsequently interpreted in the light of existing literature in the field of crisis and disaster research, post-crisis |
| Step 6 | Writing | C.S. with comments by all other authors | Manuscript preparation |
