# Supplementary material for: How well prepared are hospitals for future crises? Board members perceive their hospitals as resilient for acute crises
Source: BMC Health Serv Res. 2024 Jul 16;24:817. doi: 10.1186/s12913-024-11197-4 (PMC11251290; doi:10.1186/s12913-024-11197-4)
Supplement: Supplementary file 2 — Supplementary Material 2 [file 12913_2024_11197_MOESM2_ESM.docx]

**Appendix B**

**Interview structure and introduction script**

*General introduction and purpose of the interview*

The Covid-19 pandemic has hit healthcare to an extreme degree, creating a healthcare crisis. The organization of care processes has become more difficult to oversee due to the pandemic. Healthcare workers must continuously adapt and deal resiliently with unexpected situations to maintain patient safety. To further improve patient safety, we are working on a new approach for the healthcare organization that is based on the Safety-II idea of continuously improving work processes based on what is already going well and what can be improved (previously this was only based on what could be improved). → Safety I).

We know from research that a crisis often leads to process changes within an organization. Some organizations may even be better prepared after a crisis because they can learn from a crisis and improve processes. This is called organizational resilience. So organizational resilience is the ability of an organization (here your hospital) to “survive” a crisis and thrive in a world of uncertainty. In the context of the corona crisis, we are interested in organizational resilience within healthcare. So what about the state of resilience and what may have changed in the organizational processes.

For this purpose, I seek the conversation with you today to discuss organizational resilience in your organization. Your ideas are important to us, please share your honest opinion. There are no right or wrong answers, just different opinions. I'm going to keep an eye on the time and interrupt when necessary so we can get to all the questions.

As stated in the information letter, I will be recording the conversation so that we do not miss any important information that you give us. It is nice for the recording if one person is speaking at the same time. We will handle this recording with care and use it only for this research. The answers from this conversation will not be traced back to an individual person. We will send the final report after you, as soon as it is ready. To give permission for this, I now ask you to say out loud: 'Yes, I give permission for this'.

I would like to ask you to switch off your mobile phone.

*The structure of the interview*

In a moment, I will present you with a number of statements about organizational resilienceon which I ask you to reflect, looking back at the corona pandemic and at the future. *We used here the statements for the BRT-short form as presented in Whitman ea. 2013 [18].* The statements are divided into three topics that map thirteen indicators of resilience. I will always first explain each topic and indicator. Then I will present an accompanying statement that you will answer with “totally agree”, “agree”, “neither agree nor disagree”, “disagree”, “totally disagree”. If you don't know the answer, you can also indicate that by saying “I don't know”. Every question must be answered. I'm going to start with topic 1 now.
